# Wernicke’s encephalopathy and cranial nerve VII palsy in a 24-year-old patient with COVID-19

**DOI:** 10.1186/s12245-022-00409-5

**Published:** 2022-01-28

**Authors:** Maya Alexandri, Bradford Z. Reynolds, Hunter Smith, Bradley Michael Golden, Hartmut Gross, Jeffrey A. Switzer

**Affiliations:** 1 Department of Emergency Medicine, Augusta University Medical Center, 1120 15th Street, Augusta, GA 30912 USA; 2 Department of Neurology, Augusta University Medical Center, 1120 15th Street, Augusta, GA 30912 USA

**Keywords:** COVID-19, Non-alcohol Wernicke’s encephalopathy, Cranial nerve VII palsy

## Abstract

**Background:**

Many documented secondary neurologic manifestations are associated with COVID-19, including mild peripheral and central nervous system disorders (such as hypo/anosmia, hypo/ageusia, and cranial nerve VII palsy) and severe problems (such as ischemic stroke, Guillain-Barré syndrome, and encephalitis). The list is growing. A new addition is non-alcohol Wernicke’s encephalopathy.

**Case presentation:**

We present the case of a 24-year-old male with no past medical history who developed stroke-like symptoms two days after testing positive for COVID-19. MRI of his brain showed T2 FLAIR hyperintensity in the splenium of the corpus collosum, mamillary bodies, periaqueductal gray matter, tectum, and ventral and dorsal medulla, an MRI signal concerning for non-alcohol Wernicke’s encephalopathy. Our patient had no risk factors for Wernicke’s encephalopathy. He was admitted and started on thiamine for Wernicke’s encephalopathy and steroids for his cranial VII nerve palsy. Both his symptoms and imaging improved. He was discharged on oral thiamine. Follow-up in the Neurology Clinic has confirmed his continued stable state.

**Conclusions:**

This case is one of three documented cases of Wernicke’s encephalopathy believed to be caused by COVID-19 in patients without risk factors or chronic alcohol use. Ours is also the first case in which Wernicke’s encephalopathy presents with a concomitant cranial nerve VII palsy. While Emergency Medicine doctors must maintain a high index of suspicion for stroke in younger patients with COVID-19, our patient’s case augments the correlation between COVID-19 and Wernicke’s encephalopathy in patients without other risk factors for developing the syndrome.

## Background

The documented neurological sequelae of COVID-19 infection now encompass hypo/anosmia and hypo/ageusia, dizziness, headache, stroke, Guillain-Barré syndrome, cranial nerve VII palsy, encephalitis, and encephalopathy, including Wernicke’s encephalopathy [1–7]. This list is not exhaustive.

The mechanisms for COVID-19-related neurological impairment remain to be elucidated fully, and current hypotheses postulate both direct and indirect action of the virus on the central nervous system. The SARS-CoV-2 virus has been isolated in cerebral spinal fluid and may penetrate the brain through olfactory bulb neurons, as well as through blood-brain barrier break-down in the context of cytokine storm [5, 7]. Notwithstanding this evidence of mild central nervous system invasion in some post-mortem cases, the extent of viral infiltration does not appear to correlate with the severity of neuropathologic findings. It seems therefore more likely that neurological sequelae may be secondary to systemic inflammation, microhemorrhage, demyelination, and ischemia without direct viral invasion of neurons [1, 4, 5].

Cranial nerve VII palsy appears to be greater than the baseline incidence and more common among patients diagnosed with COVID-19 than among vaccinated people [6]. Moreover, CN VII palsy has established links with other microbial infection (e.g., HSV1, *Borrelia burgdorferi*) [4]. In the context of COVID-19 infection, CN VII palsy has been reported both as an isolated symptom and in conjunction with Guillain-Barré syndrome [2, 4, 8, 9].

Wernicke’s encephalopathy classically presents with encephalopathy, ocular symptoms, and ataxia, as a result of thiamine deficiency [1]. MRI findings include T2 FLAIR hyperintensity of the medial thalamus, dorsal medulla, tectal plate, and mammillary bodies [10]. That said, there are significant diagnostic challenges, as the paradigmatic triad is incomplete in roughly 90% of patients, thiamine deficiency may not be detected, and MRI is 53% sensitive and 93% specific [1, 10, 11]. The diagnosis is clinical and may be missed in patients who do not have a history of alcohol-use disorder [10]. Wernicke’s encephalopathy has been reported in 18 patients with COVID-19 who progressed to acute respiratory distress syndrome requiring ICU-level care [1, 12], as well as in two non-critical COVID-19 patients [5].

To our knowledge, our case report is the first instance of a patient presenting both with CN VII palsy and Wernicke’s encephalopathy in the setting of COVID-19 infection. Of additional significance, our patient makes the third documented case of Wernicke’s encephalopathy in a non-critical COVID-19 patient without other risk factors for the syndrome.

## Case presentation

A 24-year-old male with no past medical history, who had been diagnosed with COVID-19 five days previously, presented with right-sided facial droop, left-sided extremity tingling and weakness, and the sensation of falling to the left when walking. Onset of the left-sided extremity tingling and weakness and ataxia had been three days earlier; the patient had been seen at an outside hospital and prescribed gabapentin without effect. Two days later, our patient experienced right-sided facial droop and again sought emergent care. Although our patient had experienced nausea and vomiting for a week or more prior to his positive test for COVID-19, his only respiratory symptom of COVID-19 on presentation was a dry cough.

In our Emergency Department, the patient’s vital signs were: temperature 36.7°, heart rate 85 bpm, respiratory rate 20 bpm, blood pressure 139/93, and oxygen saturation 100%. Physical exam was significant for a right-sided lower motor neuron (peripheral) seventh nerve palsy, mild left-sided weakness, ataxia, and increased left-sided reflexes. He was diagnosed with CN VII palsy and started on prednisone.

**Fig. 1:**
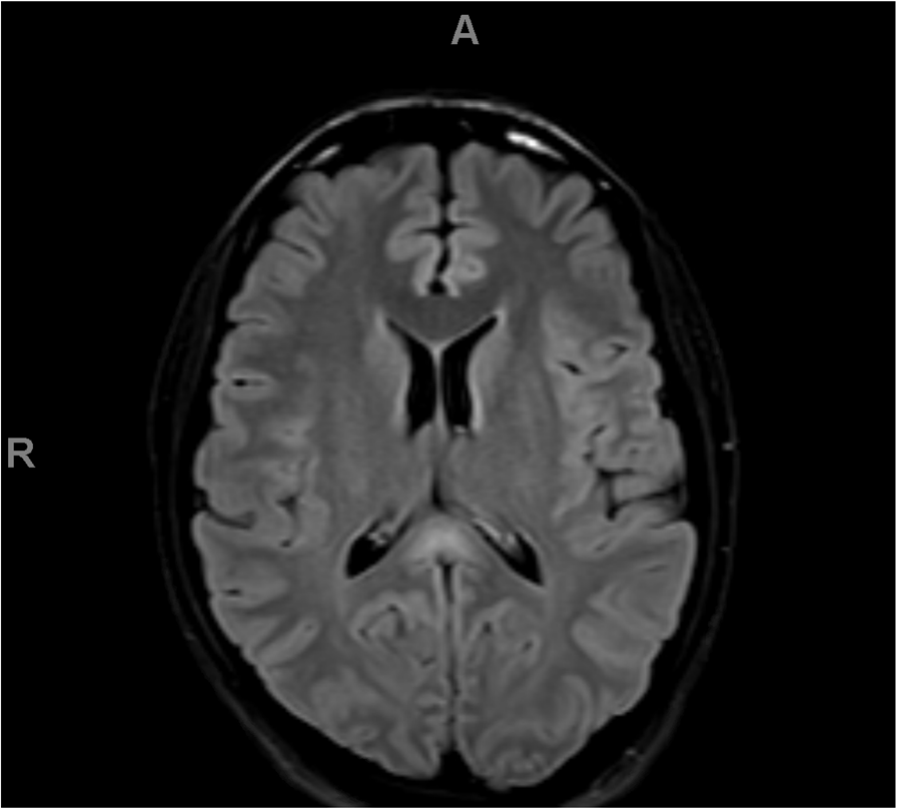
FLAIR axial images demonstrating high signal alterations in the splenium of the corpus callosum

**Fig. 2:**
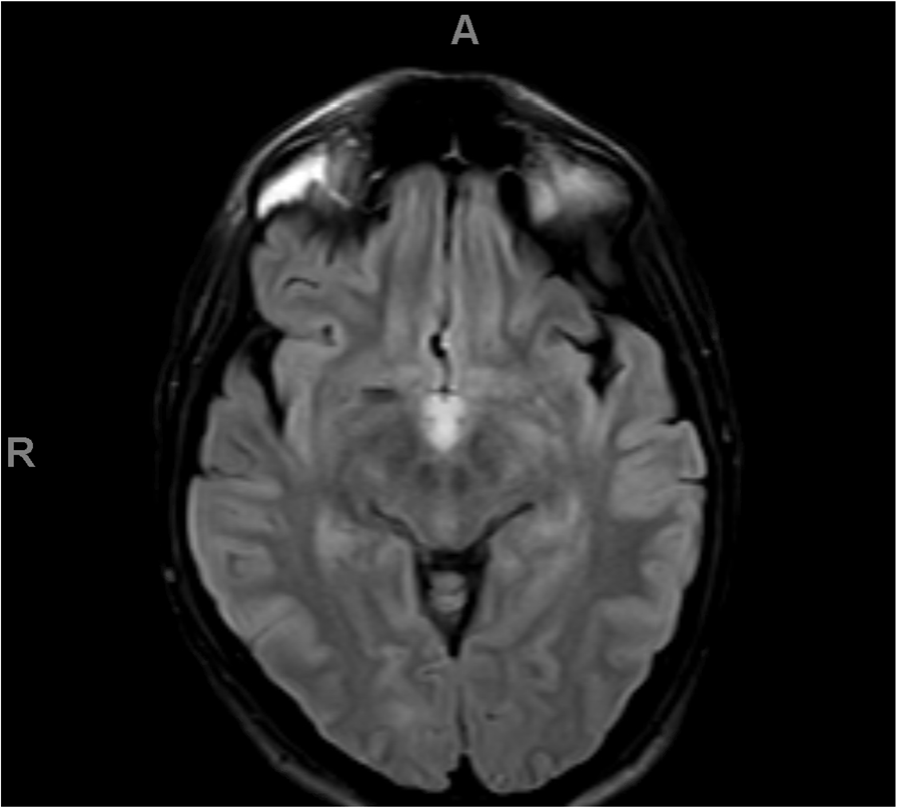
FLAIR axial images demonstrating high signal alterations in the mamillary bodies

**Fig. 3:**
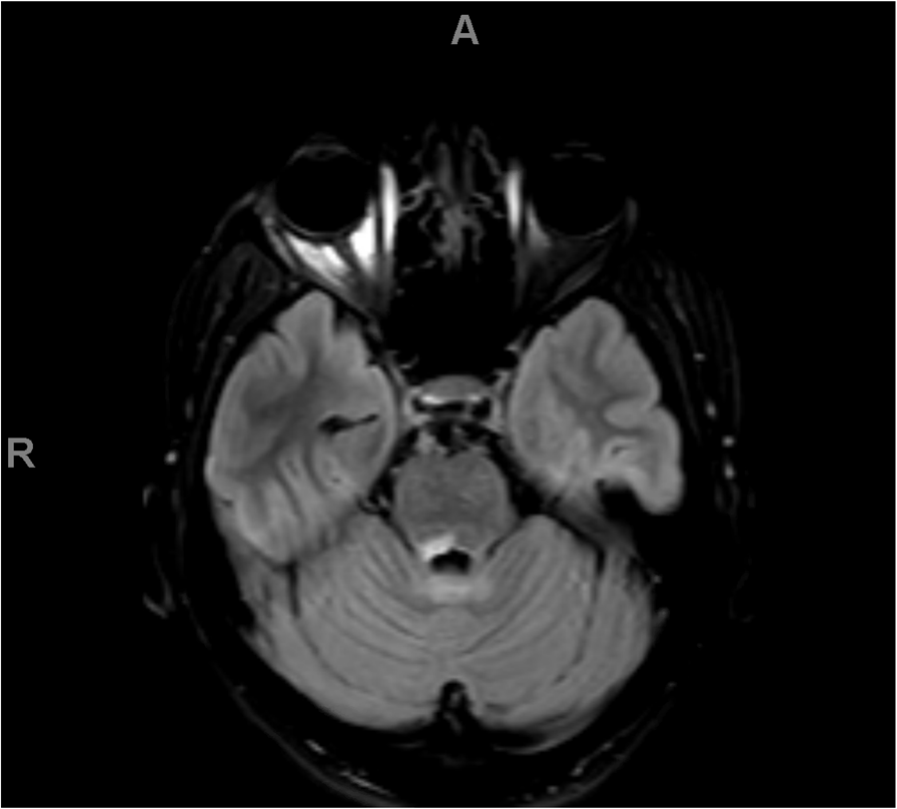
FLAIR axial images demonstrating high signal alterations in the dorsal pons

**Fig. 4:**
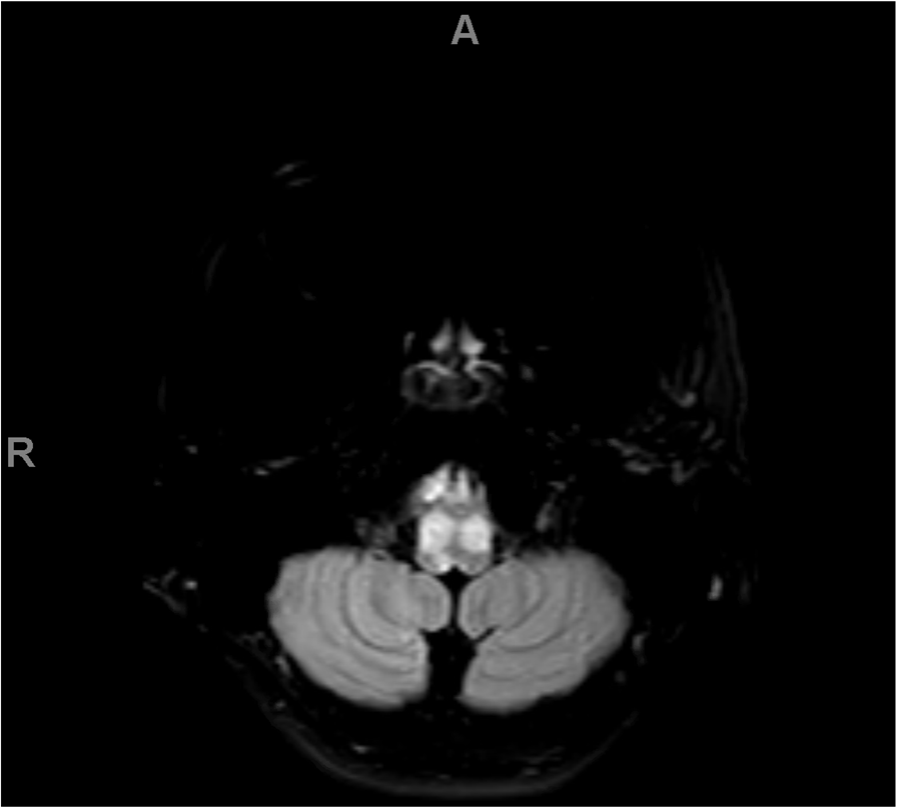
FLAIR axial images demonstrating high signal alterations in the ventral medulla

Because of the severity of his initial symptoms and the known risk of coagulopathy in patients with COVID-19, our patient also received an emergent MRI without contrast, which showed a T2 FLAIR hyperintensity in the splenium of the corpus collosum, mammillary bodies, periaqueductal gray matter, tectum, and ventral and dorsal medulla, in a pattern concerning for Wernicke's encephalopathy. See Figs. 1, 2, 3, and 4. He was admitted for workup.

While admitted, the patient received prednisone with improvement of his CN VII palsy by the next day. The patient also received 300 mg IV thiamine for 3 days, increased thereafter to 500 mg after he reported diplopia, and nystagmus was noted.

A second MRI of the brain, with and without contrast, four days after his first, showed overall improvement with significant resolution of the T2 FLAIR hyperintensity in the splenium of the corpus collosum, hypothalamus, and dorsal brainstem. Mild enhancement of the mamillary bodies and dorsal pons was present, as was an increase in the T2 FLAIR hyperintensity in the inferior olivary nuclei, which was concerning for hypertrophic olivary degeneration. An MRI of the cervical spine with contrast was unremarkable.

The balance of our patient’s workup was unrevealing. An MR angiography head without contrast was negative for stenosis, branch occlusion, or aneurysm. A transthoracic echocardiogram was negative. No lumbar puncture was performed. The patient’s thiamine level was high at 266; however, this was not collected until after he had received IV supplementation. Vitamin B12 level was high at 1369, his ALT was mildly elevated at 64, and his ferritin level was high at 406. The remainder of his labs were within normal limits, including his white blood cell count, D-dimer, ANA, rheumatoid factor, CRP, HIV, syphilis, toxicology screen, folate, and TSH.

Throughout his course, the patient’s only symptom of COVID-19 was a dry cough. His oxygen saturation on room air was within normal limits, and his chest X-ray was negative for an acute cardiopulmonary process. He was not treated for COVID-19 while inpatient.

Our patient was diagnosed with Wernicke’s encephalopathy and cranial nerve VII palsy and discharged on oral thiamine with outpatient follow-up with neurology. His condition on follow-up was stable. Informed consent for this publication was obtained.

## Discussion and conclusions

COVID-19 has distinguished itself as an infection associated with pathology of the central nervous system in younger patients [7]. The initial concern in our patient was for a posterior circulation stroke or transient ischemic attack, as the patient had crossed signs (right facial weakness and left body deficits), ataxia, and recent nausea and vomiting. However, MRI suggested a pattern more indicative of Wernicke’s encephalopathy than cerebral ischemia, and our treatment plan adjusted accordingly. Bhan et al. previously reported the case of a patient presenting with symptoms concerning for stroke who was diagnosed with Wernicke’s encephalopathy on the basis of his imaging findings [13].

Wernicke’s encephalopathy did not seem an obvious initial explanation for the patient’s clinical picture. Unlike the patient reported by Bhan, et al., who had a history of alcohol-use disorder and who had previously been treated for Wernicke’s encephalopathy, our patient is young, denied a history of chronic alcohol-use disorder, and had no known risk factors for thiamine deficiency, other than the week or so of vomiting that he experienced prior to the onset of symptoms. He did not present with an altered mental status concerning for encephalopathy, and his unilateral ocular symptoms apparent in the Emergency Department seemed well explained by CN VII palsy. CN VII palsy has only rarely been reported in the context of Wernicke’s encephalopathy [14]. The only aspect of the classic triad that our patient did exhibit was ataxia.

Nonetheless, our patient’s radiological findings of symmetric hyperintensity in the mamillary bodies, periaqueductal gray matter, and tectum are indicative of Wernicke’s encephalopathy [10]. Moreover, involvement of the splenium, dorsal medulla, and inferior olivary nuclei has been described in cases of non-alcohol related Wernicke’s encephalopathy [15]. Further investigation with a second MRI of patient’s head and an MRI of patient’s cervical spine with contrast was unrevealing of other possible diagnoses. Our patient also showed significant improvement following steroids and IV thiamine supplementation, which likewise supports the diagnosis of Wernicke’s encephalopathy.

A literature review produced two additional cases of Wernicke’s encephalopathy in patients with COVID-19, diagnosed on the basis of MRI evidence, both reported by Pascual-Goñi, et al. [5]. The first was a 60-year-old woman hospitalized for COVID-19 and exhibiting ocular symptoms of diplopia and a right-sided abducens palsy. Among her significant MRI findings were symmetrical hyperintensities in the mammillary bodies and hypothalamus.

The second patient was a 35-year-old woman with a three-week history of vomiting prior to admission, positive for COVID-19, who presented with ocular symptoms, including diplopia and bilateral abducens palsy, altered mental state, and encephalopathy. Among her significant MRI findings were symmetrical hyperintensities in the mamillary bodies and hypothalamus, though her MRI also showed limbic involvement atypical for Wernicke’s encephalopathy. That patient’s MRI a week later, following administration of thiamine, showed improvement [5].

Like these cases, our patient was positive for COVID-19, had some signs and symptoms consistent with Wernicke’s encephalopathy (e.g., diplopia, nystagmus, ataxia), and MRI findings indicative of Wernicke’s encephalopathy that improved or resolved after administration of thiamine. Pascual-Goñi and her team postulated that the concurrent presentations of two patients with COVID-19 and MRI findings concerning for Wernicke’s encephalopathy suggests a relationship between the brain pathology and the infection. Our patient contributes another instance supporting this hypothesis.

Our case demonstrates the importance of maintaining a high index of suspicion for central nervous system lesions in patients diagnosed with COVID-19, even if the viral syndrome presentation is mild or absent, and even if the patient is young. In addition, our case represents the first reported instance of CN VII palsy presenting in a COVID-19 patient in conjunction with Wernicke’s encephalopathy, rather than as a stand-alone symptom, or as a symptom of Guillian-Barré syndrome.

Our case also bolsters the literature positing a connection between Wernicke’s encephalopathy and COVID-19. At least 18 patients with COVID-19 who received ICU-level care have been reported to have developed Wernicke’s encephalopathy [1, 12], although the etiology of Wernicke’s encephalopathy in those cases appears to be related to risks of thiamine deficiency that accompany acute respiratory distress syndrome and its treatment (e.g., cytokine storm, hypercatabolic state, use of diuretics).

In cases like ours, without such risk factors, the etiology is much more difficult to hypothesize. Our patient had at least a week of poor nutrition and emesis, and this may have contributed to his susceptibility. Alternatively, thiamine-independent enzymes have been linked to the etiology of Wernicke’s encephalopathy [16]. It may be that thiamine-independent enzymes are at the root of the cerebral injury in our patient’s case, and that administration of thiamine enhanced the capacity of thiamine-dependent enzymes to compensate.

## Data Availability

Not applicable.

## Abbreviation

CN VII: Cranial nerve VII
